# MESTERIOUS RENAL MASSES

**DOI:** 10.51894/001c.122900

**Published:** 2024-08-30

**Authors:** Ross Voelker, Kathik Ranganathan, Richard Sarle

**Affiliations:** 1 Urology Residency Sparrow Hospital

## 39

### INTRODUCTION

Renal cysts are an exceedingly common finding in adults over 40. Most are simple in appearance and clearly benign. As they develop complex features like septations, calcifications, enhancement, and increased size the concern for malignancy rises. Complex Bosniak III and IV cysts are presumed malignant until proven otherwise. Cystic Nephroma and Mixed Epithelial and Stromal Tumors (MEST) are rare renal cystic neoplasms with very complex features but no malignant features. We present two cases of large complex renal masses masquerading as possible cancer that were found to be benign.

### CASE DESCRIPTION

A 62-year-old female with flank pain was found to have a 12.8cm complex right cystic mass. MRI showed innumerable enhancing internal septations and was initially thought to be a renal cell carcinoma. Due to the very large size, complete surgical removal without radical nephrectomy was not possible. The intraoperative frozen section confirmed benign pathology, and the patient had successful removal of about 75% of her mass and her kidney was spared. She had resolution of symptoms postoperatively. The final pathology was consistent with cystic nephroma. A 41-year-old female presented with an incidentally found 6cm left cystic mass. Although it was initially treated conservatively, the mass increased in size to 8cm and developed features suspicious for malignancy. The patient underwent partial nephrectomy and pathology showed MEST without malignant features.

### DISCUSSION

Cystic nephroma and MEST are related renal neoplasms with similar pathologies. Despite their complex appearance in imaging studies, they are benign tumors. They both have a heavy female predominance and can cause flank pain, hematuria, and infection. They are an important diagnosis to consider in middle aged females as over treatment for presumed malignancy could result in unnecessary radical nephrectomy.

